# Conservative Gynæcology

**Published:** 1899-08-12

**Authors:** 


### CONSERVATIVE GYNÆCOLOGY.

In his presidential address before the section of Gynæcology, Dr. Granville Bantock spoke strongly in favour of conservatism in gynæcological work. He said that for several years he had viewed with much concern a strong, and, he feared, a growing tendency, to resort to the knife too frequently instead of placing reliance upon measures of a more simple and conservative character, and, in illustration of this, he instanced the over-free use of Emmet's operation, the total removal of the generative organs in cases of fibroid of the uterus, the use of operative measures for the treatment of uncomplicated cases of retroversion of the uterus, and the removal of the appendages for the relief of minor forms of derangement of the generative apparatus. We are not sure that he is not right in some of his strictures. The fault, however, does not lie exclusively at the door of the doctors. This is an impatient generation; people—women especially—are, as they say, tired of being ill; and there is a growing demand for cure at any price. For this there is no doubt that anæsthetics, antiseptics, and the facilities for getting operations done in hospitals and nursing homes are to a large extent responsible. Neither patients nor friends really understand what is meant by an operation. An anæsthetic is given in every case. Practically the preparation is the same whether the operation be a simple or a serious one, and so, indeed, if recovery takes place, is the result. Women make no distinction. They say to themselves, "Mrs. So-and-So had an operation and got well, and why should not I?" It is no longer a matter of urging that these things should be done. Patients themselves ask to have them done, but always on the condition that if anything is done at all, and if all the trouble and expense be undertaken, the reward shall be a cure. No one cares nowadays for half measures, and the suggestion that if a simple operation does not cure there is always the major one in reserve, merely drives the patient across the street to someone else who will promise something radical. So far as the patient can be made to understand matters an operation is an operation, and there is an end of it; and it requires a strong man to risk non-success when, by making his patient run a little extra risk, success is within his grasp. Besides, he knows that his patient agrees with him, being of opinion, as so many other people have been, that "one might as well be hung for a sheep as a lamb." So conservatism is at a discount.

